# Five New Species of *Gymnopilus* from Xizang Autonomous Region of China and Surrounding Areas

**DOI:** 10.3390/jof10030220

**Published:** 2024-03-18

**Authors:** Wen-Qiang Yang, Jia-Xin Li, Mao-Qiang He, Shi-Hui Wang, Xin-Yu Zhu, Dorji Phurbu, Jian-Min Yun, Rui-Lin Zhao

**Affiliations:** 1 College of Science, Gansu Agricultural University, Lanzhou 730070, China; yangwq_107@163.com; 2 State Key Laboratory of Mycology, Institute of Microbiology, Chinese Academy of Sciences, Beijing 100101, China; lijiaxin18@mails.ucas.ac.cn (J.-X.L.);; 3 College of Life Science, University of Chinese Academy of Sciences, Beijing 100049, China; 4 Tibet Plateau Institute of Biology, Lhasa 850000, China; puduo@126.com; 5 College of Food Science and Engineering, Gansu Agricultural University, Lanzhou 730070, China

**Keywords:** agaricoid fungi, molecular phylogeny, taxonomy, hymenogastraceae

## Abstract

The species of *Gymnopilus* (Hymenogastraceae, Agricales) are commonly recognized as wood-decaying fungi. Certain members of this genus have been identified as psilocybin-producing mushrooms. *Gymnopilus* exhibits a diverse range and has a global distribution. In this study, a total of seventy-eight specimens were gathered from ten provinces in China. A comprehensive molecular phylogenetic analysis was conducted, employing gene sequences including ITS, nrLSU, nrSSU, *rpb1*, *rpb2*, and *tef1-α*. Additionally, morphological examinations were also carried out. The phylogenetic topology of *Gymnopilus* from this study generally agreed with previous studies and facilitated the identification of all those specimens. As a result, eleven species, including five newly discovered ones named *Gy. gyirongensis*, *Gy. variisporus*, *Gy. tomentosiceps*, *Gy. tenuibasidialis*, and *Gy. aurantipileatus*, were recognized. Significantly, four of the five newly identified species are native to the Xizang Autonomous Region, emphasizing their specialization in this distinctive habitat. This research contributes to our comprehension of *Gymnopilus* diversity and lays the groundwork for the conservation and sustainable utilization of *Gymnopilus* resources.

## 1. Introduction

The genus *Gymnopilus* P. Karst. (Hymenogastraceae, Agaricales), which was originally described in Sweden in 1879, has more than 200 species in the world [1,2,3]. The species of *Gymnopilus* are commonly known as wood-decaying fungi in forests and play the role of decomposers in the cycle of matter [1,4]. *Gymnopilus* species can be characterized by the morphological characteristics of their hemispherical or convex cap; yellowish, brownish, greenish, or purplish pileus; adnate or adnexed lamellae; fibrous and sometimes membranous or filamentous curtain; the presence of an arachnoid to membranous veil; roughened or verrucose appearance; and basidiospores that are yellow to yellowish brown and then rusty brown. Some species usually have a bitter taste and clamp connections present on almost all kinds of hyphae [5,6,7,8,9]. The majority of *Gymnopilus* species are saprobes, and some species generate psilocybin, a psychedelic compound that can lead to nerve poisoning [7,10].

Romagnesi (1942) first divided the genus into two groups: *Annulatae* Romagn. (1942: 89) and *Cortinatae* Romagn. (1942: 89). Group *Annulatae* is characterized by having a persistent, membranous annulus, while in the other group, the annulus is absent. Hesler (1969) and Singer (1986) accepted those two groups of *Gymnopilus* and put this genus under Cortinariaceae, while Kühner (1980) and Guzmán-Dávalos et al. (2003) classified it under Strophariaceae. Kirk et al. (2008) considered it to belong to Hymenogastraceae [1,2]. Later, Guzmán-Dávalos (1995) established a section, *Macrospori* Guzm.-Dáv (1995: 119) to *Gymnopilus*, which contains species characterized by large spores and a lack of an annulus [7,11]. Recently, morphological examination combined with molecular phylogenetic analysis has been widely used to study this genus and describe new species from different parts of the world [2,12,13,14,15,16,17,18,19,20].

The taxonomic study of *Gymnopilus* started relatively late in China, and Shu-Qun Deng (1963) was the first person to record *Gymnopilus* species from China in his monumental work *Fungi of China*. Recently, Li (2012) reported a total of thirty *Gymnopilus* species, including six new species from the tropical region [7,21]. Liu (2019) reported a species from northeast China, and she also constructed a phylogenetic tree of *Gymnopilus* based on ITS sequences. This phylogenetic tree, which was identical to those of previous studies, showed six stable clades [5,7,12]. In terms of geographic distribution, most *Gymnopilus* species were found in parts of southern China, such as the Guangdong, Yunnan, Sichuan, and Hainan provinces, and some were from northeastern China (Jilin province). Generally, the species of *Gymnopilus* in China is poorly reported, and up to now, only thirty-one species have been recorded [7,21].

In this study, we collected seventy-eight *Gymnopilus* specimens from ten provinces of China and two from Thailand. Molecular phylogenetic analysis combined with morphological examination revealed they belong to eleven species, of which five are proposed as new species.

## 2. Materials and Methods

### 2.1. Specimen Collection and Morphological Study

We collected specimens in the field from the Xizang Autonomous Region and surrounding provinces of China and Thailand. The macroscopic features of the collected specimens were photographed and recorded, along with their odor and the fresh specimens’ changes in color upon injury. The specimens were dried completely overnight at 55 °C using a food desiccator, sealed in plastic bags, and deposited in the Herbarium Mycologicum Academiae Sinicae, Beijing, China (HMAS).

The anatomical and cytological features, including lamellae, pileipellis, basidiospores, basidia, and cystidia, of the dried specimens were observed following the protocols described in [22]. For microscopic characterization, the sections were mounted in 5% KOH solution. Thirty measurements of mature spores were taken for each specimen, along with twenty measurements of basidia and cystidia, which included x, the mean of length by width ± SD; Q, the quotient of basidiospore length to width; and Qm, the mean of Q-values ± SD [23]. The hymenial cystidia and pileocystidia were stained with 1% aqueous Congo red solution [24]. The color designation referred to methuen handbook of colour.

### 2.2. DNA Extraction, PCR Amplification, and Sequencing

Genomic DNA was extracted from 5 to 10 mg of dried specimen using a Broad-spectrum Plant Rapid Genomic DNA Kit (Biomed, Beijing, China) and preserved at −20 °C.

We used primers ITS4 and ITS5 [25] for the internal transcribed spacer (ITS) region of the nuclear ribosomal DNA repeat, primers LROR and LR5 [26] for the Large Subunit (LSU) region, primers NS1 and NS4 [27] for the nuclear nrSSU-rDNA region, primers EF1-983F and EF1-1567R [28] for translation elongation factor alpha (*tef1-α*), and RNA polymerase II gene (*rpb2*) with 6F/7CR [29] and primers Af/Cr for RNA polymerase II (largest subunit) (*rpb1*) [30]. Genes were amplified by polymerase chain reaction (PCR) using the procedures mentioned in [26,28,29,31]. The PCR products were sent to a commercial biotech company (Baimaide Biotechnique Company, Beijing, China) for sequencing.

### 2.3. Molecular Phylogenetic Study

The sequences produced from this study and some generated by a previous work and deposited in the NCBI GenBank database were used in our phylogenetic analyses [2,7,9,19,20,32,33] (Table 1). Sequences of multigene data were aligned separately using Muscle version 3.6 [34], then manually adjusted to remove ambiguous regions in BioEdit version 7.0.4 [35]. The six partitions were assembled in PhyloSuite v1.2.2 [36] in the order of six loci (ITS, nrLSU, nrSSU, *rpb1*, *rpb2*, *tef1-α*). Maximum likelihood (ML) analysis was performed using RAxmlGUI 1.3 under a GTRGAMMA model with one thousand rapid bootstrap (BS) replicates [37]. Bayesian Inference (BI) analysis was performed using MrBayes v3.2.6 [38]. Six Markov chains were run for 2,000,000 generations, and trees were sampled every 100th generation. Burn-ins were determined in Tracer version 1.6 with an ESS value higher than 200, and the remaining trees were used to calculate Bayesian posterior probabilities (PP). The trees were displayed in Fig Tree version 1.4.0 [39].

A total of fifty-nine ITS sequences and four nrLSU sequences of *Gymnopilus* were downloaded from GenBank for phylogenetic analysis. *Galerina marginata* (Batsch) Kühner was selected as the outgroup. The newly generated sequence samples from this study have been deposited in GenBank.

The resulting file after tree construction was used to view the phylogenetic tree using iTOL [40]. Bootstrap Support (BS) ≥ 70% was considered significantly supported. Bayesian posterior probability (PP) ≥ 0.90 was regarded as significant.

## 3. Results

### 3.1. Molecular Phylogenetic Analysis Results

**Figure 1:**
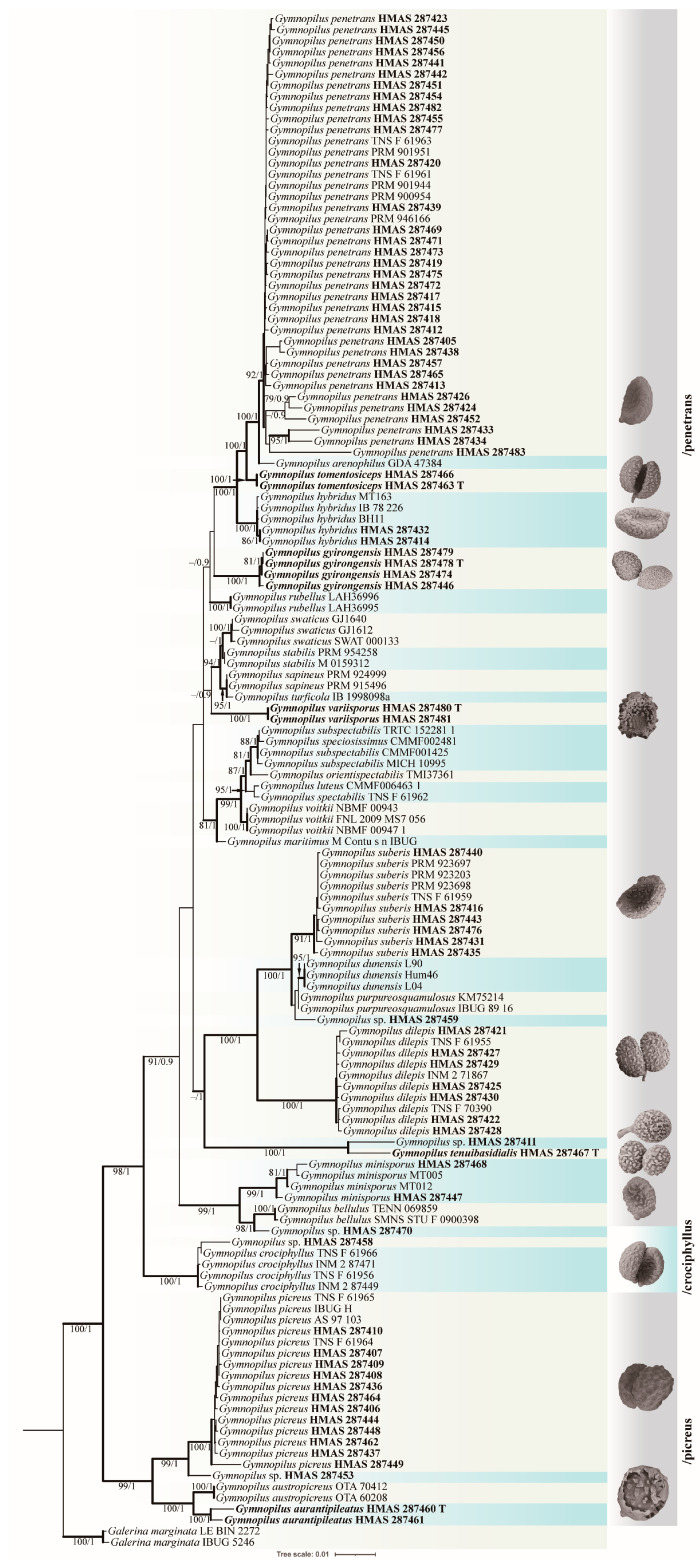
Maximum Likelihood (ML) phylogram of *Gymnopilus* inferred from partial ITS, nrLSU, nrSSU, *rpb1*, *rpb2*, and *tef1-α* sequences. The tree is rooted by *Ga. marginata*. Maximum Likelihood support values (>70) and posterior probabilities (>0.90) are shown on the branch (ML/PP). The sequences produced from this study are in bold. “T” refers to the sequences from the types of specimens in this study.

The seventy-eight specimens of *Gymnopilus* collected in this study, which represented eleven species, were included in the phylogenetic analyses with the outgroup species *Galerina marginata*. During our study, seventy-six ITS sequences, seventy-three nrLSU sequences, seventy-five nrSSU sequences, fifteen *rpb1* sequences, sixty *rpb2* sequences, and sixteen *tef1-α* sequences were newly generated in this study (Table 1). There was a total of five thousand and thirty-one bp (base pairs) in the final alignment after assembling those six gene sequences, of which eight hundred and ninety-one characters are from nrLSU, five hundred and forty-four characters are from tef1-α, seven hundred and six characters are from rpb2, one thousand three hundred and twenty-seven characters are from rpb1, eight hundred and ninety characters are from nrSSU, and six hundred and seventy-three characters are from ITS. The phylogenetic tree of the ML and MrBayes topology were generally the same using those six gene sequences. The Maximum Likelihood tree is shown in Figure 1, with bootstrap and posterior probability values indicated on the branches.

### 3.2. Taxonomy

**Figure 2:**
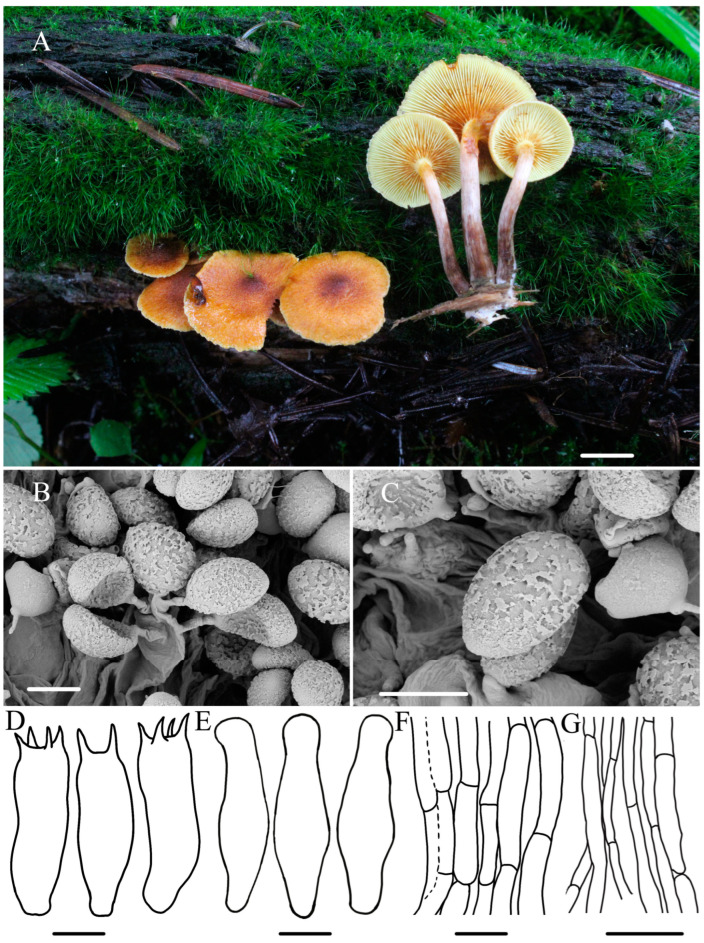
The morphology of *Gymnopilus gyirongensis* sp. nov. of holotype *ZRL20220779* (HMAS 287478). (**A**) Basidiomata; (**B**,**C**) basidiospores; (**D**) basidia; (**E**) cheilocystidia; (**F**) pileipellis; (**G**) stipitipellis. Scale bar = 1 cm for (**A**); 5 μm for (**B**–**D**); 4 μm for (**E**); 5 μm for (**F**,**G**).

***Gymnopilus gyirongensis*** R.L. Zhao and W.Q. Yang, sp. nov., Figure 2.

**Fungal Names:** FN 571665.

**Etymology:** —‘gyirong’ refers to the location where the type specimen was collected.

***Typification*:** China, Xizang Autonomous Region, Shigatse, Gyirong County, Gyirong Town, N 28°23′45″, E 85°23′34″, 3441 m asl, 2 August 2022, collected by *Rui-Lin Zhao* and *Xin-Yu Zhu*, *ZRL20220779* (holotype HMAS 287478).

***Diagnosis*:** Pileus medium-sized with orange–yellow to brownish orange, covered with yellowish rust squamules and umbonate center; basidiospores medium-sized (6.2–6.8 × 3.4–4.3) μm with ornamentation moderately developed; scattered or gathered on mossy wood with rotting pine needles.

**Macroscopic description:** Pileus 12–30 mm diameter, plano-convex to plane, with a low broad umbo, orange-–yellow (4–6AB4–6) to brownish orange (5–6BC4–5), slightly wavy to serrate margins, moist surface covered with yellow and rust squamules, denser and darker in the middle of the cap. Context thin, pale white (1A1) to pale yellow (2B3), color unchanging upon cutting. Lamellae adnate to slightly adnexed, pale yellow (4A3) to yellowish brown (4B8), with a few ferruginous spots (7B6), crowded, unequal, L = 0.7–1.3 cm, I = 4.8–6.1 mm. Stipe 2.8–3.6 × 0.2–0.5 cm, cylindrical hollow, sometimes thickening at the base, fibrillose, pale white (1A1) from to the top and brown (7E7) from center to the base, color changing to yellowish brown (4B8), base with whitish or cream mycelium. Odor indistinct.

**Microscopic description:** Basidiospores (5.9–)6.2–6.8(–7.0) × (2.9–)3.4–4.3(–4.5) μm, Q = 1.6–1.8, avQ = 1.7, broadly ellipsoid, distinct suprahilar depression in side view, ornamentation moderately developed, germ pore absent. Basidia (18.5–)19.5–21.4(–21.8) × 5.1–6.0 μm, narrowly clavate, mostly four-sterigmate, rarely two-sterigmate, sterigmata 2.2–3.5 μm long, hyaline in KOH, oil-like droplets present. Cheilocystidia 21.2–26.8 × 4.8–6.8 μm, lecythiform to narrowly utriform, cylindrical, narrowly clavate, capitate to sub-capitate, hyaline, oil droplets present, thin-walled, well-defined basal clamp. Pleurocystidia not observed. Pileipellis is a cutis of filamentous hyphae, 3.6–11 μm diam, with very light rusty brown plasmatic pigment, thin-walled. Stipitipellis filamentous hyphae 4.4–7.5 μm diam, some inflated hyphae observed with constrictions at septa, thin-walled, clamp connections present. A yellowish pigment dissolves when lamellae are mounted in KOH.

**Habitat:** —Scattered or clustered on mossy, damp wood with rotting pine needles.

**Other material examined:** China, Xizang Autonomous Region, Shigatse, Gyirong County, Gyiron Town, N 28°23′45″, E 85°23′34″, 3441 m asl, 2 August 2022, collected by *Rui-Lin Zhao* and *Xin-Yu Zhu*, *ZRL20220790* (HMAS 287479); China, Xizang Autonomous Region, Nyingchi City, Forest ecological monitoring station, N 29°39′2″, E 94°42′58″, 3880 m asl, 27 July 2021, collected by *Bin Cao* and *Xin-Yu Zhu*, *ZRL20211058* (HMAS 287474); China, Sichuan Province, Tibetan Autonomous Prefecture of Garzê, Yajiang County, Gexigou National Nature Reserve, 15 August 2020, collected by *Rui-Lin Zhao* and *Xi-Xi Han*, *ZRL20200269* (HMAS 287446).

**Notes:** *Gymnopilus gyirongensis* sp. nov. can be easily distinguished by its orange-yellow to orange–brown pileus covered with yellowish rust squamules and umbonate center. Microscopically, this new species has medium-sized spores with moderately developed ornamentation. In the field, another proposed new species, *Gy. tomentosiceps*, may be confused with *Gy. gyirongensis* due to the fact that they both have yellowish caps; however, they have different habitats: *Gy. tomentosiceps* usually grows on mossy, soil under coniferous forest trees, but *Gy. gyirongensis* prefers fruiting on damp wood with rotting pine needles. In this study, phylogenetic analysis demonstrated that four specimens of this new species formed a cohesive cluster with full support. Additionally, they were positioned closely to *Gy. penetrans*, *Gy. hybridus*, and *Gy. tomentosiceps* (Figure 1). *Gymnopilus penetrans* is similar to *Gy. gyirongensis* because they share the characteristic of a smooth pileus covered with yellow and rusty brown squamules on the surface and can be found under conifers and deciduous trees [2,32]. However, *Gy. penetrans* develop bigger basidiopores (7.2–9.9 × 4–5.5 μm) than those of *Gy. gyirongensis* [6]. Moreover, these two species have 29 base pair differences in their ITS sequences. The four ITS sequences of this new species display four or five base pair differences. Another similar species, *Gy. hybridus*, can easily be distinguished from this species due to it having bigger basidiospores (6–8 × 4–4.5 μm) and basidia (20–28 × 5–8 μm) [5,7,41].

**Figure 3:**
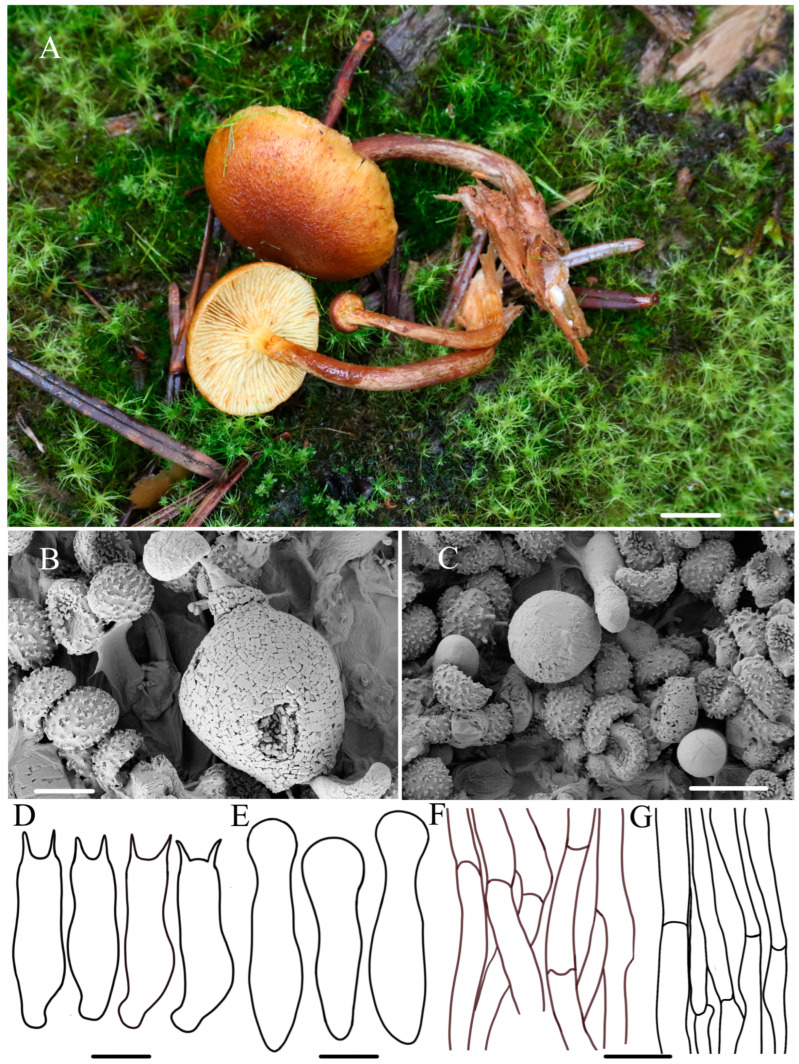
The morphology of *Gy. variisporus* sp. nov. of holotype *ZRL20220827* (HMAS 287480). (**A**). Basidiomata; (**B**,**C**) basidiospores; (**D**) basidia; (**E**) cheilocystidia; (**F**) pileipellis; (**G**) stipitipellis. Scale bar = 1 cm for (**A**); 5 μm for (**B**–**D**); 4 μm for (**E**); 5 μm for (**F**,**G**).

***Gymnopilus variisporus*** R.L. Zhao and W.Q. Yang, sp. nov., Figure 3.

**Fungal Names:** FN 571764.

**Etymology:** —‘variisporus’ refers to variable ornamentation on the spore surface during maturity.

***Typification*:** China, Xizang Autonomous Region, Shigatse, Gyirong County, Gyirong Town, N 28°23′45″, E 85°23′34″, 3441 m asl, 2 August 2022, collected by *Mao-Qiang He* and *Bin Cao*, *ZRL20220827* (holotype HMAS 287480).

***Diagnosis*:** Pileus medium-sized, covered with yellow to rusty squamules; stipe yellowish brown to rusty brown; basidiospores 6.7–7.3 × 4.5–5.1 μm, golden brown to yellow–brown; scattered or clustered in meadows on moist ground of decaying pine needles.

**Macroscopic description:** Pileus 4–39 mm across, convex to plano-convex when young, becoming hemispherical to plano-convex or slightly concaved at maturity, yellow–brown (4B8), smooth in dried specimens, glabrous, with inflexed to involute margin, moist surface covered with yellow and rusty squamules. Context thin but thicker at the center, pale white (1A1) to yellowish brown (4B8), unchanging upon cutting. Lamellae adnate to slightly adnexed, crowded to close, pale yellow (4A3) to yellowish brown (4B8), and finally ferruginous with spots (7B6). Stipe 2.9–6.3 × 0.2–0.7 cm, cylindrical, hollow, fibrous, yellowish brown to rusty brown (2B3–5C5), base with white mycelium, lacking veil. Odor indistinct.

**Microscopic description:** Basidiospores (6.5–)6.7–7.3(–7.6) × (3.8)4.5–5.1(–5.4) μm, Q = 1.43–1.49, avQ = 1.46, ellipsoid, distinct suprahilar depression in side view, ornamentation moderately developed, not more than 0.2 μm high, golden brown to yellow–brown. Basidia 14.1–16.9 × 4.3–5.0 μm, clavate, colorless, with oil droplets or pigment, sterigmata 1.6–2.3 μm long with yellowish brown. Cheilocystidia 14.1–18.0 × 5.2–5.6 μm, utriform to narrowly lageniform, some cylindrical to narrowly clavate, hyaline, with obtuse or sub-capitate apex, base pedunculate or pedicellate, non-gut, well-defined basal clamp. Pleurocystidia absent. Pileipellis cutis hyphae 4–7 (8.5) µm in diameter, septate, crowded, branched, clamp connection present, non-guttulated, thin-walled. Stipitipellis cutis hyphae 6.5–9 µm in diameter, branched, filamentous, clamp connection present. A yellowish pigment dissolves when lamellae are mounted in KOH.

**Habitat:** —Scattered or clustered in meadows on moist ground of decaying pine needles, characterized by the absence of bark and cork, summer.

**Other material examined:** China, Xizang Autonomous Region, Shigatse, Gyirong County, Gyirong Town, N 28°23′45″, E 85°23′34″, 3441 m asl, 2 August 2022, collected by *Mao-Qiang He* and *Bin Cao*, *ZRL20220830* (HMAS 287481).

**Notes:***Gymnopilus variisporus* sp. nov. is distinctive in having a hemispherical to plano-convex pileus covered with yellowish squamules and possessing ellipsoid spores with moderate ornamentation. In the field, *Gy. variisporus* has relatively similar features to *Gy. gyirongensis* sp. nov. because they both possess yellowish pileus covered with rust squamules, but *Gy. variisporus* has bigger basidiospores (6.7–7.3 × 4.5–5.1 μm). Phylogenetic analyses in this study showed that *Gy. variisporus* formed a distinct lineage with fully supported values and was a sister to *Gy. swaticus*, *Gy. stabilis*, and *Gy. sapineus* (Figure 1). The three known species have bigger basidiospores and different habitats compared to this new species. Their respective characterization are *Gy. stabilis* having yellowish orange to light orange pilei and possessing bigger basidispores (7.5–8.5 × 4.5–5 µm), growing in sandy soil [32]; *Gy. sapineus* has yellowish brown to rusty brown pileus covered with fibrillose-tomentum to tomentum–scaly, and it possess bigger basidiospores (7.3–8.2 × 4.5–5.2 µm), fruiting on dead wood, but *Gy. variisporus* usually scatter or cluster in meadows, specifically on moist ground [6,32]; *Gy. swaticus* has velutinous to slightly tomentose and pileus and bigger basidiospores (8.6–10.0 × 4.5–5.6 μm), growing on *Piceae smithiana* [2,19].

**Figure 4:**
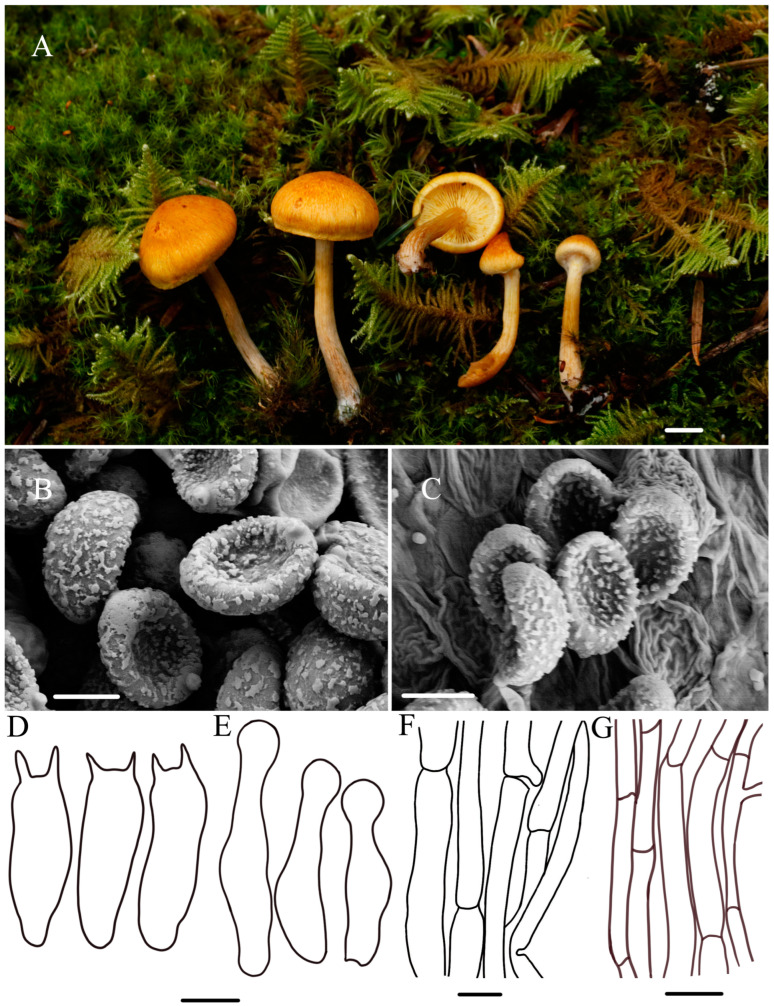
The morphology of *Gy. tomentosiceps* sp. nov. of holotype *ZRL20210594* (HMAS 287463). (**A**). Basidiomata; (**B**,**C**) basidiospores; (**D**) basidia; (**E**) cheilocystidia; (**F**) pileipellis; (**G**) stipitipellis. Scale bar = 1 cm for (**A**); 5 μm for (**B**,**C**); 6 μm for (**D**,**E**); 1 μm for (**F**); 1 μm for (**G**).

***Gymnopilus tomentosiceps*** R.L. Zhao and W.Q. Yang, sp. nov., Figure 4.

**Fungal Names:** FN 571765.

**Etymology:** —‘tomentosiceps’ means the pileus of this species is covered with tomentose scales.

***Typification*:** China, Xizang Autonomous Region, Zayü County, N 28°36′46″, E 98°5′22″, 4110 m asl, 21 July 2021, collected by *Rui-Lin Zhao*, *Ming-Yu Zhu*, and *Bin Cao*, *ZRL20210594* (holotype HMAS 287463).

***Diagnosis*:** Pileus medium-sized, covered with tomentose scales; basidiospores 5.5–6.8 × 3.8–4.5 μm, coarsely roughened with large and irregular wart; cheilocystidia present, 21.2–26.8 × 4.8–6.8 μm, lecythiform to narrowly utriform, capitate to sub-capitate.

**Macroscopic description:** Pileus 8–26 mm diameter, hemispherical, pale yellow (4A3) to darker ochre–yellow (6C8), with undulating margin, covered with tomentose scales. Context thin, pale white (1A1) to yellowish brown (4B8), unchanging upon cutting. Lamellae adnate to slightly adnexed, pale yellow (4A3) to yellowish brown (4B8), with ferruginous spots and slightly wrinkled in dried specimens, glabrous, crowded, unequal, L = 0.7–1.3 cm, I = 4.8–6.1 mm. Stipe 2.4–3.9 × 0.2–0.4 cm, cylindrical, hollow, sometimes tapering at base, pearl white to brown (3B1-7E7), base with or without whitish or cream mycelium. Pleats yellow to rusty brown. Odor indistinct.

**Microscopic description:** Basidiospores (4.6–)5.5–6.8(–7.1) × (3.7–)3.8–4.5(–4.8) μm, Q = 1.45–1.51 μm, aveQ = 1.48 μm, ellipsoid, distinct suprahilar depression in side view, slightly thickened wall, coarsely roughened with large and irregular wart, germ pore absent, yellowish brown. Basidia 14.5–19.1 × 4.5–5.0 μm, narrowly clavate, hyaline, oil-like droplets present, two to four sterigmata, sterigmata 2.8–3.6 μm long. Cheilocystidia 21.2–26.8 × 4.8–6.8 μm, lecythiform to narrowly utriform, capitate to sub-capitate, hyaline, oil droplets present, thin-walled, well-defined basal clamp. Pileipellis cutis hyphae 2.7–7.1 µm in diameter, septate, crowded, branched, clamp connection present, non-guttulated, thin-walled. Stipitipellis hyphae 1.2–4.8 µm in diameter, branched, filamentous, clamp connection present. A yellowish pigment dissolves when lamellae are mounted in KOH.

**Habitat:** —Grows on mossy, moist soil under coniferous forest trees.

**Other material examined:** China, Xizang Autonomous Region, Bomi County, N 29°47′30″, E 95°41′50″, 3670 m asl, 24 July 2021, collected by *Xin-Yu Zhu*, *Ming-Zhe Zhang*, *ZRL20210795* (HMAS 287466).

**Notes:** *Gymnopilus tomentosiceps* sp. nov. is easily distinguished by its pileus, which is completely covered with a pale yellow tinge. In the field, *Gy. tomentosiceps* is morphologically similar to *Gy. orientispectabilis* because it possesses minutely fibrillose–scaly, but *Gy. orientispectabilis* has bigger basidiospores (7.2–9.0 × 4.8–6 μm) and basidia (22.2–36.0 × 6.6–9.6 μm) [20]. In the phylogenetic tree (Figure 1), *Gy. penetrans* and *Gy. arenophilus* are sisters to *Gy. tomentosiceps*, consistent with previous studies [2,7,41]. Morphologically, they can be easily distinguished. *Gymnopilus penetrans* has finely innately rusty ochre to rusty brown fibrillose-striped pileus and moderately developed ornamentation in terms of the ellipsoidal spores, growing on the dead wood of conifers and deciduous trees, and all of those are different from *Gy. tomentosiceps*. [2,32]. *Gy. arenophilus* differs by its bigger basidiospores (8.5–10.5 × 5.5–6.5 μm) and longer basidia (26–35 × 7–9 μm). Additionally, it exhibits distinct growing conditions, being found on sandy soil and occasionally attached to wood chips or charcoal [14].

**Figure 5:**
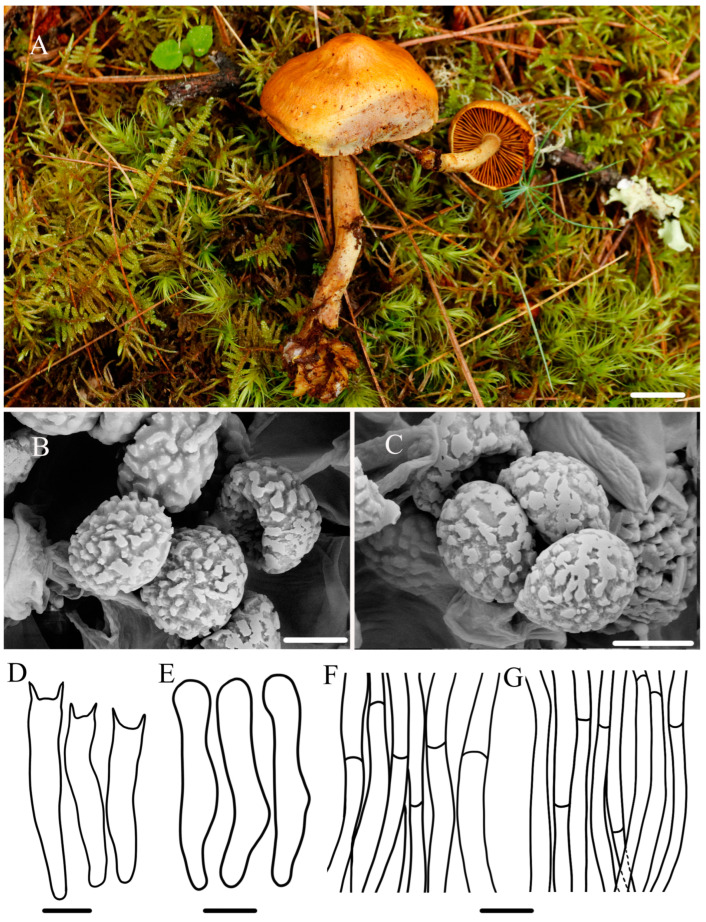
The morphology of *Gy. tenuibasidialis* sp. nov. of holotype *ZRL20210911* (HMAS 287467). (**A**). Basidiomata; (**B**,**C**) basidiospores; (**D**) basidia; (**E**) cheilocystidia; (**F**) pileipellis; (**G**) stipitipellis. Scale bar = 1 cm for (**A**); 5 μm for (**B**,**C**); 6 μm for (**D**); 4 μm for (**E**); 5 μm for (**F**,**G**).

***Gymnopilus tenuibasidialis*** R.L. Zhao and W.Q. Yang, sp. nov., Figure 5.

**Fungal Names:** FN 571766.

**Etymology:** —‘tenuibasidialis’ refers to the shape of the basidia of this species (narrow and long).

***Typification*:** China, Xizang Autonomous Region, Nyingchi City, Bomi County, Spruce forest, N 29°52′50″, E 95°34′43″, 2700 m asl, 26 July 2021, collected by *Xin-Yu Zhu* and *Ming-Zhe Zhang*, *ZRL20210911* (holotype HMAS 287467).

***Diagnosis*:** Pileus medium-sized, abrupt papilla, yellowish to mustard with brown patches of varying sizes; basidiospores 4.8–5.1 × 3.7–4.0 μm, broadly ellipsoid; cheilocystidia present, 30.1–35.5 × 3.7–4.8 μm, slender.

**Macroscopic description:** Pileus 18–34 mm diameter, with abrupt papilla, yellowish to mustard (4B6–5C8), surface fibrillose, brown patches of varying sizes. Context thin, pale white (1A1), color unchanging upon cutting. Lamellae 4–6 mm broad, yellowish brown (4B8), adnate to sinuate, straight, sparser, unequal in length, edge entire or slightly serrulate, lamellulae mostly in two tiers. Stipe 1.3–5.7 × 0.2–0.6 cm, yellowish brown (4B8), cylindrical, hollow, fibrillose, dull surface, equal in thickness at the top and bottom. Odor indistinct.

**Microscopic description:** Basidiospores (4.1–)4.8–5.1(–5.6) × (3.5–)3.7–4.0(–4.2) μm, Q = 1.28–1.30 μm, broadly ellipsoid, with broadly rounded apices, moderately roughened with irregular warts and short ridges, non-dextrinoid to obscurely dextrinoid. Basidia 21.4–24.0 × 3.6–4.8 μm, four-spored, sterigmata 1.8–5.1 μm long, clavate to cylindrical, oil-like droplets present, usually constricted near or above the middle. Cheilocystidia 30.1–35.5 × 3.7–4.8 μm, lecythiform to narrowly utriform, capitate to sub-capitate but occasionally without a swollen apex, hyaline, well-defined basal clamp. Pleurocystidia not observed. Pileipellis cutis hyphae 3.8–13.2 µm in diameter, septate, crowded, branched, clamp connection present, non-guttulated, thin-walled. Stipitipellis hyphae 1.4–7.4 µm in diameter, branched, filamentous, clamp connection present. A yellowish pigment dissolves when lamellae are mounted in KOH.

**Habitat:** —Single or scattered under pines, usually in a wet environment.

**Other material examined:** No other specimens.

**Notes:** *Gymnopilus tenuibasidialis* sp. nov. is easily distinguished by possessing abrupt papill pileus with brown patches and presenting slender cheilocystidia (30.1–35.5 × 3.7–4.8 μm). In the phylogenetic tree (Figure 1), *Gy. tenuibasidialis* formed a distinct lineage separated from other *Gymnopilus* species with fully supported lineages. *Gymnopilus purpureosquamulosus*, *Gy. dunensis*, *Gy. suberis*, and *Gy. dilepis* were close to this new species in phylogeny; however, they can be easily distinguished by macro- and micro-morphology. *Gymnopilus dilepis* can differ by its convex to plano-convex pileus with ruby to violet brown squamules, bigger basidiospores (6–7.5 × 4.8–6 µm), shorter and thicker cheilocystidia (20–30.2 × 8–12 µm) [12,42]. *Gymnopilus purpureosquamulosus* differs in having brown–purple to purple pileus with a central depression, a surface covered with reddish violet squamules and lamellae that turn grey with KOH and FeSO_4_, and bigger basidiospores (7–8 × 4–5.5 µm) [18]. *Gymnopilus dunensis* can differ by its pileus, covered with greyish squamules when young; bigger basidiospores (8.5–10.7 × 4.9–6.6 μm) and basidia (24.9–29.4 × 8.5–9.7 μm); and the fact that it is gregarious in small groups on sandy soil [19]. *Gymnopilus suberis* can differ by its pileus (initially cream to white before finally tunin sulfur yellow), a fibrous stipe with a cortinoid veil, and bigger basidiospores (7.2–8.8 × 4.4–4.8 µm) and basidia (24–27 × 6.5 µm) [17]. Phylogenetically, an unidentified specimen represented by *ZRL20151336* (HMAS 287411) is a sister to this new species; however, they exhibit large differences in spore ornamentation (Figure 1).

**Figure 6:**
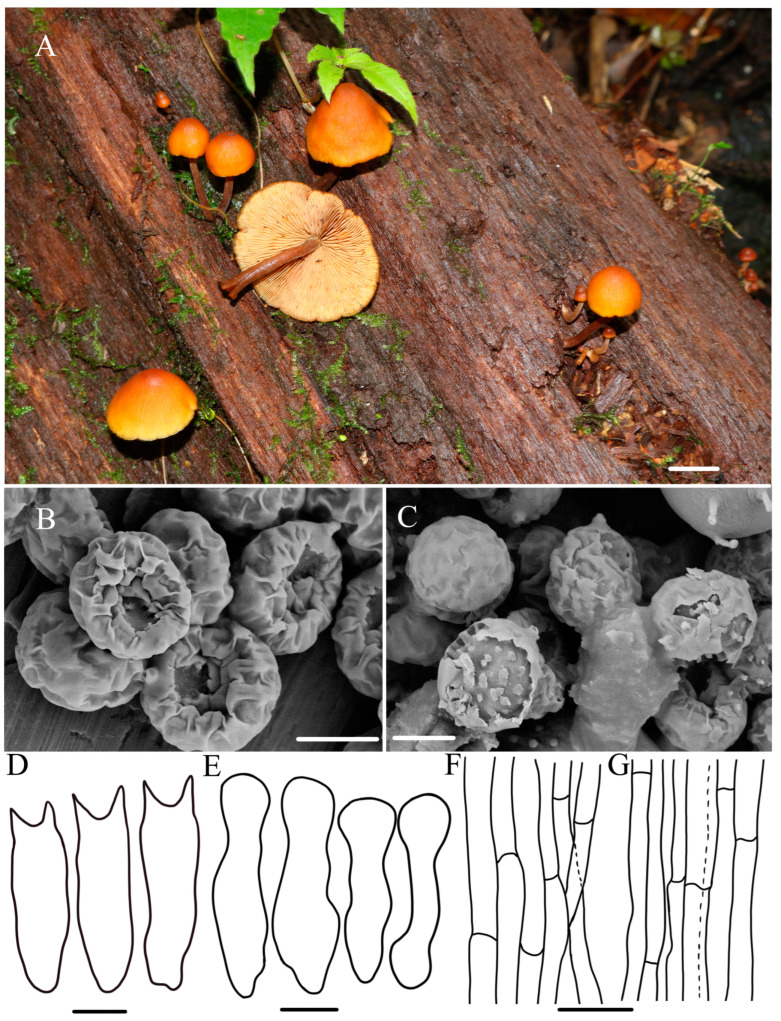
The morphology of *Gy. aurantipileatus* sp. nov. of holotype *ZRL20210361* (HMAS 287460). (**A**). Basidiomata; (**B**,**C**) basidiospores; (**D**) basidia; (**E**) cheilocystidia; (**F**) pileipellis; (**G**) stipitipellis. Scale bar = 1 cm for (**A**); 4 μm for (**B**,**C**); 5 μm for (**D**); 3 μm for (**E**); 3 μm for (**F**,**G**).

***Gymnopilus aurantipileatus*** R.L. Zhao and W.Q. Yang, sp. nov., Figure 6.

**Fungal Names:** FN 571767.

**Etymology:** —‘aurantipileatus’ means the pileus color of the type of specimen is orange.

***Typification*:** China, Yunnan Province, Jingdong Yi Autonomous County, Ailao Mountains, N 23°36′–24°56′, E 100°44′–101°30′, 3166 m asl, 4 July 2021, collected by *Rui-Lin Zhao*, *Mao-Qiang He*, *Min-Zhe Zhang*, *Xin-Yu Zhu*, and *Mei-Qi Wang*, *ZRL20210361* (HMAS 287460).

***Diagnosis*:** Pileus medium-sized, with lemon yellow to orange yellow, not scaly; stipe 0.7–1.9 × 0.1–0.3 cm, yellowish brown; basidiospores 5.7–6.5 × 4.5–5.3 μm, ornamentation developed; scattered or clumped on damp wood.

**Macroscopic description:** Pileus 2–30 mm diameter, hemispherical, lemon yellow (4A8) to orange–yellow (5A8), surface smooth and glabrous, not scaly. Context thin, yellowish brown (4B8), unchanging upon cutting. Lamella adnate, pale yellow (4A3), close to crowded, with ferruginous patches, edge even. Stipe 0.7–1.9 × 0.1–0.3 cm, moderately yellowish brown (4B8), hollow, terete, equal or widened to the base, context moist, with spots of the same color as the lamella and gradually becomes denser near the cap. Odor indistinct.

**Microscopic description:** Basidiospores (5.3–)5.7–6.5(–6.8) × (4.3–)4.5–5.3(–6.0) μm, Q = 1.2–1.3 μm, broadly ellipsoid, verrucose with small to medium warts, coarsely roughened with large and irregular warts, ornamentation developed, golden brown to yellow-brown. Basidia 19.4–23.1 × 5.4–7.0 μm, four- or two-spored, clavate, colorless, with oil droplets or pigment, sterigmata 2.3–4.2 μm long, yellow–brown with occasional individual sterigmata yellowish brown. Cheilocystidia 9–16.5 × 3–4.8 μm, clavate, lageniform to narrowly lageniform with capitate apex, yellowish brown. Pleurocystidia not seen. Pileipellis cutis hyphae 2.4–6.7 µm in diameter, septate, crowded, branched, clamp connection present, non-guttulated, thin-walled. Stipitipellis hyphae 3.2–6.3 µm in diameter, branched, filamentous, clamp connection present. A yellowish pigment dissolves when lamellae are mounted in KOH.

**Habitat:** —Scattered or in clusters overgrown with wood, usually in a wet environment.

**Other material examined:** China, Yunnan Province, Jingdong Yi Autonomous County, Ailao Mountains, N 23°36′–24°56′, E 100°44′–101°30′, 3166 m asl, 4 July 2021, collected by *Rui-Lin Zhao*, *Mao-Qiang He*, *Ming-Zhe Zhang*, *Xin-Yu Zhu*, and *Mei-Qi Wang*, *ZRL20210364* (HMAS 287461).

**Notes:** *Gymnopilus aurantipileatus* sp. nov. is easily distinguished by having smooth to glabrous pileus and small cheilocystidia (9–16.5 × 3–4.8 μm). In the field, *Gy. aurantipileatus* sp. nov. is similar to *Gy. purpureosquamulosus*, because they both have pileus broadly convex to plano-convex, light orange to orange–yellow. *Gymnopilus picreus* and this new species have the type of same habitat, dead coniferous trees. However, *Gy. picreus* is distinguished by its squamulose pileus, rusty stained lamellae, dark brown stipe with white pulverulence, and bigger basidiospores (7.0–9.5 × 4.5–6.0 μm) [7]. Compared to *Gy. crociphyllus*, which has oxide yellow to light brown pileus and sometimes mustard yellowish smudges, bigger basidia (27.8–30.9 × 6.3–7.5 μm) and bigger cheilocystidia (23.3–26.9 × 6.7–8.7 μm) [43], *Gy. aurantipileatus* sp. nov. is also different. *Gy. austropicreus* differs by featuring a pale lemon-yellow pileus and lamellae that darken to black with the application of KOH. Furthermore, it possesses bigger basidiospores (6.6–9 × 4.5–6 µm) and cheilocystidia (15–27 × 4–6 µm) [44].

## 4. Discussion

Based on a combination of morphological and phylogenetic analyses, seventy-eight specimens were identified as eleven *Gymnopilus* species. Six of them are known species, and they are *Gy. penetrans* (Fr) Murrill, *Gy. hybridus* (Gillet) Maire, *Gy. suberis* (Maire) Singer, *Gy. dilepis* (Berk. & Broome) Singer, *Gy. minisporus* (T. Bau & M. T. Liu), and *Gy. picreus* (Pers.) P. Karst. These known species have been reported in tropical and subtropical areas based on previous studies [2,4,5,6,7,8,42,45], and in this study, we found them in ten provinces. Five new species—*Gy. gyirongensis* sp. nov.; *Gy. tomentosiceps* sp. nov.; *Gy. variisporus* sp. nov.; *Gy. aurantipileatus* sp. nov.; *Gy. tenuibasidialis* sp. nov.—mainly from the Xizang Autonomous Region and surrounding provinces of China have been described.

In this study, the macroscopic and microscopic morphology of those five new species are described in detail. Some macro- and micro-differences can be used to separate them from each other. *Gymnopilus tomentosiceps* is characterized by having a small and tomentose cap, while *Gy. tenuibasidialis* has an abrupt papilla cap. The mature spores of these five new species are all distinct, with suprahilar depressions in side view, but the shapes and ornamentation on the surface (Figure 2, Figure 3, Figure 4, Figure 5 and Figure 6) are different among these species. The basidiospores of *Gy. variisporus* and *Gy. tomentosiceps* are ellipsoid, while those of the other species are broadly ellipsoid; *Gy. aurantipileatus* has developed ornamentation on basidiospores, while the ornamentation of *Gy. variisporus* and *Gy. aurantipileatus* is very variable in the process of maturity (Figure 3 and Figure 6). In general, even though these five new species have similar macro characteristics, their characteristics in terms of spore shape and ornamentation are distinct and different from each other, as supported by our molecular phylogenetic analysis.

In the phylogenetic tree (Figure 1), twenty-nine included species of *Gymnopilus* segregate into three distinct clades (/*penetrans*, /*crociphyllus*, and /*picreus*), and these three clades are part of the six clades delineated in a previous study and are phylogenetically positioned in the same location [7,12]. In the /*penetrans* clade, *Gy. hybridus* is regarded as a synonym of *Gy. penetrans* by Holec (2005) [6]. However, in Europe, *Gy. penetrans* is classified as a singular species, with *Gy. hybridus* considered as a synonym [44,45], or as distinct and formed as two separate species (*Gy. penetrans* and *Gy. hybridus*) [46], and these species differ in color, the development of the veil, the presence of rusty spots on lamellae, and the shape of cystidia [6]. In our phylogenetic tree (Figure 1), *Gy. tomentosiceps*, *Gy. hybridus*, and *Gy. penetrans* show a close relationship but in different phylogenetic positions. Furthermore, a total of 21 nucleotide differences were detected in the ITS region between *Gy. tomentosiceps* and *Gy. penetrans*, and 24 nucleotide differences were detected between *Gy. tomentosiceps* and *Gy. hybridus*. Thus, those three species are identified as separate species. Differences in macro characteristics exist: *Gy. prnetrans* typically exhibits smooth or slightly wrinkled caps and stipe with white fibrillose partial veil remnants; *Gy. hybrids* feature hygrophanous streaks, as well as smooth and scale-free caps; and *Gy. tomentosiceps* is characterized by a cap covered with tomentose scales. *Gymnopilus crociphyllus* (Cooke and Massee) Pegler has distinctive characteristics, such as a fasciculate fruiting body, large pileus size, and a rumpled pileus margin [43]. In the */picreus* clade, which includes *Gy. austropicreus* B.J. Rees and *Gy. aurantipileatus* sp. nov, this clade can be morphologcially characterized as having the same ornamentation on spores encased in a membrane which will gradually fall off with maturity. In another study [46], *Gy. picreus* (FT.) P. Karsten and *Gy. austropicreus* always emerged basal to the remainder of the species in the phylogenetic tree, which could refer to how they may be ancestral to other species in the genus [47]. *Gymnopilus picreus* groups grow as a saprophytes on the dead wood of conifers (mostly *Picea abies*; less frequently *Pinus sylvestris*) and, rarely, deciduous trees (*Betula*, *Fagus sylvatica*) [6], but other *Gymnopilus* species could grow on dead wood or on mulch-rich soil [2]. Thus, we suspect that the rationale for the types of wood rot they seek for nutrition is possibly ancestral in this genus.

In China, a total of thirty-one *Gymnopilus* species, including seven species originally described to be from China, have been published so far [7,9,21]. *Gymnopilus* is a species-rich genus, and the previous studies from China included several provinces, such as the Guangdong, Yunnan, Sichuan, Hainan, and Jilin provinces. In this paper, we have reported species from more parts of China, namely the Xizang Autonomous Region, resulting in a total of thirty-six species of *Gymnopilus* that have been recorded in China. Furthermore, four of the five new species are originally from the Xizang Autonomous Region, which indicates the special nature of the species, as they are from such a unique habitat. This study complements our understanding of *Gymnopilus* diversity and lays a foundation for the conservation and utilization of related *Gymnopilus* resources.

## Figures and Tables

**Table 1:** Information of the sequences generated from this study. Missing sequences are indicated by “-”.

Species	Specimen-Voucher	Country	ITS	LSU	SSU	*rpb1*	*rpb2*	*tef1-* *α*	Source
***Gymnopilus penetrans***	**HMAS 287405**	**China: Yunnan**	**-**	**OR915134**	**OR915221**	**-**	**PP058997**	**-**	This study
***Gy. penetrans***	**HMAS 287412**	**China: Zhejiang**	**OR913484**	**OR915135**	**OR915178**	**PP210943**	**PP058994**	**PP165679**	This study
***Gy. penetrans***	**HMAS 287413**	**China: Zhejiang**	**OR913499**	**OR915128**	**OR915204**	**-**	**-**	**-**	This study
***Gy. penetrans***	**HMAS 287415**	**China: Xizang Autonomous Region**	**OR913483**	**OR915122**	**OR915208**	**PP210941**	**PP058992**	**PP165677**	This study
***Gy. penetrans***	**HMAS 287417**	**China: Xizang Autonomous Region**	**OR913494**	**OR915118**	**OR915190**	**PP210948**	**PP058998**	**PP165675**	This study
***Gy. penetrans***	**HMAS 287418**	**China: Xizang Autonomous Region**	**OR913504**	**OR915130**	**OR915174**	**-**	**-**	**PP165678**	This study
***Gy. penetrans***	**HMAS 287419**	**China: Xizang Autonomous Region**	**OR913497**	**OR915117**	**OR915179**	**PP210937**	**-**	**PP165676**	This study
***Gy. penetrans***	**HMAS 287423**	**China: Gansu**	**OR913486**	**OR915114**	**OR915222**	**-**	**-**	**-**	This study
***Gy. penetrans***	**HMAS 287424**	**China: Yunnan**	**OR982115**	**OR915132**	**OR915180**	**PP210947**	**PP058990**	**PP165668**	This study
***Gy. penetrans***	**HMAS 287426**	**China: Yunnan**	**OR982116**	**OR915133**	**OR915188**	**PP210946**	**PP058996**	**PP165669**	This study
***Gy. penetrans***	**HMAS 287433**	**China: Yunnan**	**OR982119**	**OR915125**	**OR915173**	**-**	**PP059007**	**-**	This study
***Gy. penetrans***	**HMAS 287434**	**China: Yunnan**	**OR982120**	**OR915126**	**OR915193**	**-**	**PP059009**	**-**	This study
***Gy. penetrans***	**HMAS 287438**	**China: Sichuan**	**OR913507**	**OR915140**	**OR915201**	**-**	**PP058989**	**-**	This study
***Gy. penetrans***	**HMAS 287439**	**China: Sichuan**	**OR913506**	**OR915129**	**OR915210**	**PP210940**	**PP058988**	**-**	This study
***Gy. penetrans***	**HMAS 287441**	**China: Sichuan**	**OR913496**	**OR915112**	**OR915176**	**-**	**PP059002**	**-**	This study
***Gy. penetrans***	**HMAS 287442**	**China: Sichuan**	**OR913489**	**OR915119**	**OR915177**	**-**	**PP059000**	**PP165674**	This study
***Gy. penetrans***	**HMAS 287450**	**China: Sichuan**	**OR913501**	**OR915124**	**OR915192**	**-**	**PP059003**	**-**	This study
***Gy. penetrans***	**HMAS 287451**	**China: Sichuan**	**OR913493**	**OR915127**	**OR915195**	**-**	**PP059004**	**PP165680**	This study
***Gy. penetrans***	**HMAS 287452**	**China: Yunnan**	**OR913524**	**-**	**OR915220**	**-**	**-**	**-**	This study
***Gy. penetrans***	**HMAS 287445**	**China: Sichuan**	**OR913485**	**OR915121**	**OR915191**	**-**	**PP059005**	**-**	This study
***Gy. penetrans***	**HMAS 287454**	**China: Sichuan**	**OR913508**	**OR915131**	**OR915182**	**PP210942**	**PP058999**	**-**	This study
***Gy. penetrans***	**HMAS 287455**	**China: Sichuan**	**OR913495**	**OR915139**	**OR915189**	**-**	**PP059008**	**PP165681**	This study
***Gy. penetrans***	**HMAS 287456**	**China: Sichuan**	**OR913492**	**OR915115**	**OR915197**	**-**	**PP059001**	**-**	This study
***Gy. penetrans***	**HMAS 287457**	**China: Sichuan**	**OR913500**	**OR915137**	**OR915214**	**PP210939**	**PP058993**	**-**	This study
***Gy. penetrans***	**HMAS 287465**	**China: Xizang Autonomous Region**	**OR913505**	**OR915120**	**OR915183**	**PP210944**	**PP059006**	**-**	This study
***Gy. penetrans***	**HMAS 287469**	**China: Xizang Autonomous Region**	**OR913488**	**OR915141**	**OR915206**	**PP210950**	**PP058987**	**PP165673**	This study
***Gy. penetrans***	**HMAS 287482**	**China: Hubei**	**OR913502**	**-**	**-**	**-**	**-**	**-**	This study
***Gy. penetrans***	**HMAS 287483**	**China: Hubei**	**OR982114**	**OR976241**	**-**	**-**	**-**	**PP165671**	This study
***Gy. penetrans***	**HMAS 287452**	**China: Yunnan**	**OR913524**	**-**	**OR915220**	**-**	**-**	**-**	This study
***Gy. penetrans***	**HMAS 287471**	**China: Xizang Autonomous Region**	**OR913503**	**OR915138**	**OR915185**	**PP210949**	**PP058986**	**PP165682**	This study
***Gy. penetrans***	**HMAS 287472**	**China: Xizang Autonomous Region**	**OR913487**	**OR915113**	**OR915203**	**PP210951**	**PP058995**	**-**	This study
***Gy. penetrans***	**HMAS 287473**	**China: Xizang Autonomous Region**	**OR913491**	**OR915123**	**OR915199**	**PP210945**	**PP058985**	**PP165670**	This study
***Gy. penetrans***	**HMAS 287475**	**China: Xizang Autonomous Region**	**OR913498**	**OR915116**	**OR915186**	**PP210938**	**PP058991**	**PP165683**	This study
***Gy. penetrans***	**HMAS 287477**	**China: Chongqing**	**OR913490**	**OR915136**	**OR915196**	**-**	**PP059010**	**PP165672**	This study
*Gy. penetrans*	TNS-F-61961	Japan	KT368684	**-**	**-**	**-**	**-**	**-**	Kasuya et al. (2016)
*Gy. penetrans*	TNS-F-61963	Japan	KT368685	**-**	**-**	**-**	**-**	**-**	Khan et al. (2017)
*Gy. penetrans*	PRM 901944	Czech R.	MW750184	**-**	**-**	**-**	**-**	**-**	Holec et al. (2021)
*Gy. penetrans*	PRM 900954	Czech R.	MW750186	**-**	**-**	**-**	**-**	**-**	Holec et al. (2021)
*Gy. penetrans*	PRM 901951	Czech R.	MW750185	**-**	**-**	**-**	**-**	**-**	Holec et al. (2021)
*Gy. penetrans*	PRM 946166	Poland	MW750183	**-**	**-**	**-**	**-**	**-**	Holec et al. (2021)
*Gy. hybridus*	MT163	China	MK036417	**-**	**-**	**-**	**-**	**-**	Liu and Bau (2019)
*Gy. hybridus*	IB-78-226	Sweden	AF501548	**-**	**-**	**-**	**-**	**-**	Liu and Bau (2019)
*Gy. hybridus*	BH11	USA	MF773630	**-**	**-**	**-**	**-**	**-**	Liu and Bau (2019)
***Gy. hybridus***	**HMAS 287432**	**China: Heilongjiang**	**OR913479**	**-**	**-**	**-**	**-**	**-**	This study
***Gy. hybridus***	**HMAS 287414**	**China: Heilongjiang**	**OR913478**	**OR915144**	**OR915200**	**-**	**PP059011**	**-**	This study
*Gy. suberis*	PRM-923698	Czech Republic	HG969653	**-**	**-**	**-**	**-**	**-**	Liu and Bau (2019)
*Gy. suberis*	PRM-923697	Czech Republic	HG969652	**-**	**-**	**-**	**-**	**-**	Holec et al. (2016)
*Gy. suberis*	PRM-923203	Spain	HG969654	**-**	**-**	**-**	**-**	**-**	Holec et al. (2016)
*Gy. suberis*	TNS-F-61959	Japan	KT368689	**-**	**-**	**-**	**-**	**-**	Khan et al. (2017)
***Gy. suberis***	**HMAS 287416**	**China: Xizang Autonomous Region**	**OR913522**	**OR915107**	**OR915209**	**-**	**PP059023**	**-**	This study
***Gy. suberis***	**HMAS 287435**	**China: Sichuan**	**OR913520**	**OR915106**	**OR915194**	**-**	**PP059021**	**-**	This study
***Gy. suberis***	**HMAS 287440**	**China: Sichuan**	**OR913523**	**OR915103**	**OR915211**	**-**	**-**	**-**	This study
***Gy. suberis***	**HMAS 287431**	**China: Inner Mongolia Autonomous Region**	**OR913521**	**OR915108**	**OR915175**	**-**	**PP059020**	**-**	This study
***Gy. suberis***	**HMAS 287443**	**China: Sichuan**	**OR913518**	**OR915105**	**OR915181**	**-**	**PP059024**	**-**	This study
***Gy. suberis***	**HMAS 287476**	**China: Yunnan**	**OR913519**	**OR915104**	**OR915186**	**-**	**PP059022**	**-**	This study
***Gy. suberis***	**HMAS 287421**	**Thailand: Nan**	**OR913511**	**OR915098**	**OR915225**	**-**	**PP059016**	**-**	This study
***Gy. suberis***	**HMAS 287422**	**Thailand: Nan**	**OR913512**	**OR915099**	**OR915205**	**-**	**PP059014**	**-**	This study
***Gy. suberis***	**HMAS 287425**	**China: Yunnan**	**OR913509**	**OR915100**	**OR915223**	**-**	**PP059018**	**-**	This study
***Gy. suberis***	**HMAS 287427**	**China: Yunnan**	**OR913515**	**OR976239**	**OR976274**	**-**	**-**	**-**	This study
***Gy. suberis***	**HMAS 287428**	**China: Guangxi**	**OR913514**	**OR915101**	**OR915226**	**-**	**PP059017**	**-**	This study
***Gy. suberis***	**HMAS 287429**	**China: Guangxi**	**OR913513**	**OR976240**	**OR915224**	**-**	**-**	**-**	This study
***Gy. suberis***	**HMAS 287430**	**China: Guangxi**	**OR913510**	**OR976238**	**OR915227**	**-**	**PP059015**	**-**	This study
*Gy. dilepis*	INM-2-71867	Japan	KT368680	**-**	**-**	**-**	**-**	**-**	Khan et al. (2017)
*Gy. dilepis*	TNS-F-61955	Japan	KT368681	**-**	**-**	**-**	**-**	**-**	Khan et al. (2017)
*Gy. dilepis*	TNS-F-70390	Japan	KU727215	**-**	**-**	**-**	**-**	**-**	Kasuya et al. (2016)
***Gy. picreus***	**HMAS 287406**	**China: Xizang Autonomous Region**	**OR913466**	**OR915146**	**OR915171**	**-**	**PP058976**	**-**	This study
***Gy. picreus***	**HMAS 287407**	**China: Zhejiang**	**OR913470**	**OR915145**	**OR915164**	**-**	**-**	**-**	This study
***Gy. picreus***	**HMAS 287408**	**China: Zhejiang**	**OR913471**	**OR915147**	**OR915168**	**-**	**PP058970**	**-**	This study
***Gy. picreus***	**HMAS 287409**	**China: Zhejiang**	**OR913461**	**OR915149**	**OR915172**	**-**	**PP058971**	**-**	This study
***Gy. picreus***	**HMAS 287410**	**China: Zhejiang**	**OR913469**	**OR915148**	**OR915165**	**-**	**PP058969**	**-**	This study
***Gy. picreus***	**HMAS 287436**	**China: Sichuan**	**OR913462**	**OR915157**	**OR915169**	**-**	**PP058972**	**-**	This study
***Gy. picreus***	**HMAS 287437**	**China: Sichuan**	**OR913463**	**-**	**OR915161**	**-**	**PP058968**	**-**	This study
***Gy. picreus***	**HMAS 287448**	**China: Sichuan**	**OR913467**	**OR915154**	**OR915162**	**-**	**PP058974**	**-**	This study
***Gy. picreus***	**HMAS 287449**	**China: Sichuan**	**OR913465**	**OR915153**	**OR915167**	**-**	**PP058973**	**-**	This study
***Gy. picreus***	**HMAS 287464**	**China: Xizang Autonomous Region**	**-**	**OR915151**	**OR915170**	**-**	**PP058977**	**-**	This study
***Gy. picreus***	**HMAS 287462**	**China: Xizang Autonomous Region**	**OR913468**	**OR915152**	**OR915163**	**-**	**PP058978**	**-**	This study
***Gy. picreus***	**HMAS 287444**	**China: Sichuan**	**OR913464**	**OR915155**	**OR915166**	**-**	**PP058975**	**-**	This study
*Gy. picreus*	TNS-F-61965	Japan	**KT368687**	**-**	**-**	**-**	**-**	**-**	Kasuya et al. (2016)
*Gy. picreus*	TNS-F-61964	Japan	**KT368686**	**-**	**-**	**-**	**-**	**-**	Kasuya et al. (2016)
*Gy. picreus*	IBUG-H	Finland	**AY281003**	**-**	**-**	**-**	**-**	**-**	Holec et al. (2021)
*Gy. picreus*	AS 97-103	Australia	**AF501557**	**-**	**-**	**-**	**-**	**-**	Rees et al.. (2002)
***Gy. minisporus***	**HMAS 287468**	**China: Xizang Autonomous Region**	**OR913517**	**-**	**OR915228**	**-**	**-**	**-**	This study
***Gy. minisporus***	**HMAS 287447**	**China: Sichuan**	**OR913516**	**OR915096**	**OR915212**	**-**	**PP058982**	**-**	This study
*Gy. minisporus*	MT005	China	MK036415	**-**	**-**	**-**	**-**	**-**	Liu and Bau (2019)
*Gy. minisporus*	MT012	China	MK036416	**-**	**-**	**-**	**-**	**-**	Liu and Bau (2019)
***Gy.* sp. **	**HMAS 287470**	**China: Xizang Autonomous Region**	**OR913480**	**OR915097**	**OR915219**	**-**	**PP058981**	**-**	This study
***Gy.* sp. **	**HMAS 287453**	**China: Sichuan**	**OR913460**	**OR915150**	**OR915213**	**-**	**PP058967**	**-**	This study
***Gy.* sp. **	**HMAS 287458**	**China: Yunnan**	**OR982118**	**OR915109**	**OR915215**	**-**	**-**	**-**	This study
***Gy.* sp. **	**HMAS 287459**	**China: Yunnan**	**OR982117**	**OR915102**	**OR915216**	**-**	**PP059019**	**-**	This study
***Gy.* sp. **	**HMAS 287411**	**China: Zhejiang**	**OR913457**	**OR915094**	**OR915207**	**-**	**PP058979**	**-**	This study
***Gy. aurantipileatus***	**HMAS 287460**	**China: Yunnan**	**OR913458**	**OR915110**	**OR915159**	**-**	**PP058965**	**-**	This study
***Gy. aurantipileatus***	**HMAS 287461**	**China: Yunnan**	**OR913459**	**OR915111**	**OR915160**	**-**	**PP058966**	**-**	This study
***Gy. tomentosiceps***	**HMAS 287463**	**China: Xizang Autonomous Region**	**OR913481**	**OR915142**	**OR915217**	**-**	**PP059012**	**-**	This study
***Gy. tomentosiceps***	**HMAS 287466**	**China: Xizang Autonomous Region**	**OR913482**	**OR915143**	**OR915184**	**-**	**PP059013**	**-**	This study
***Gy. tenuibasidialis***	**HMAS 287467**	**China: Xizang Autonomous Region**	**OR913456**	**OR915095**	**OR915218**	**-**	**PP058980**	**-**	This study
***Gy. gyirongensis***	**HMAS 287478**	**China: Xizang Autonomous Region**	**OR913476**	**OR976246**	**OR976275**	**-**	**-**	**-**	This study
***Gy. gyirongensis***	**HMAS 287479**	**China: Xizang Autonomous Region**	**OR913477**	**OR976245**	**OR976276**	**-**	**-**	**-**	This study
***Gy. gyirongensis***	**HMAS 287474**	**China: Xizang Autonomous Region**	**OR913475**	**OR915156**	**OR915198**	**-**	**PP058984**	**-**	This study
***Gy. gyirongensis***	**HMAS 287446**	**China: Sichuan**	**OR913474**	**OR976244**	**OR915202**	**-**	**PP058983**	**-**	This study
***Gy. variisporus***	**HMAS 287480**	**China: Xizang Autonomous Region**	**OR913472**	**OR976242**	**OR976272**	**-**	**-**	**-**	This study
***Gy. variisporus***	**HMAS 287481**	**China: Xizang Autonomous Region**	**OR913473**	**OR976243**	**OR976273**	**-**	**-**	**-**	This study
*Gy. sapineus*	PRM 924999	Czech R.	MW750187	**-**	**-**	**-**	**-**	**-**	Holec et al. (2021)
*Gy. sapineus*	PRM 915496	Czech R.	MW750188	**-**	**-**	**-**	**-**	**-**	Holec et al. (2021)
*Gy. stabilis*	PRM 954258	Czech R.	MW750182	**-**	**-**	**-**	**-**	**-**	Holec et al. (2021)
*Gy. stabilis*	M 0159312	Germany	MW750189	**-**	**-**	**-**	**-**	**-**	Holec et al. (2021)
*Gy. subspectabilis*	TRTC 152281	Canada	MN206898	**-**	**-**	**-**	**-**	**-**	Holec et al. (2021)
*Gy. subspectabilis*	CMMF001425	Canada	MN206902	**-**	**-**	**-**	**-**	**-**	Thorn et al. (2020)
*Gy. subspectabilis*	MICH 10995	USA	MN206901	**-**	**-**	**-**	**-**	**-**	Thorn et al. (2020)
*Gy. swaticus*	SWAT 000133	Pakistan	MF149864	MF149865	**-**	**-**	**-**	**-**	R. Khurshid et al. (2023)
*Gy. swaticus*	GJ1640	Pakistan	MF149866	MF149867	**-**	**-**	**-**	**-**	R. Khurshid et al. (2023)
*Gy. swaticus*	GJ1612	Pakistan	MF149863	**-**	**-**	**-**	**-**	**-**	Holec et al. (2021)
*Gy. turficola*	IB 1998098a	Norway	AF325669	**-**	**-**	**-**	**-**	**-**	Holec et al. (2021)
*Gy. voitkii*	NBM-F00947	Canada	MN206872	**-**	**-**	**-**	**-**	**-**	Thorn et al. (2020)
*Gy. voitkii*	FNL 2009 MS7-056	Canada	MN206879	**-**	**-**	**-**	**-**	**-**	Thorn et al. (2020)
*Gy. voitkii*	NBM-F00943	Canada	MN206867	**-**	**-**	**-**	**-**	**-**	Thorn et al. (2020)
*Gy. arenophilus*	GDA-47384	Spain	EU518421	**-**	**-**	**-**	**-**	**-**	Khan et al. (2017)
*Gy. maritimus*	M.Contu s.n. IBUG	Italy	EU518419	**-**	**-**	**-**	**-**	**-**	Guzmán-Dávalos et al. (2009)
*Gy. orientispectabilis*	TMI37361	Japan	MN206910	**-**	**-**	**-**	**-**	**-**	Thorn et al. (2020)
*Gy. speciosissimus*	CMMF002481	Canada	MN206895	**-**	**-**	**-**	**-**	**-**	Thorn et al. (2020)
*Gy. luteus*	CMMF006463	Canada	MN206889	**-**	**-**	**-**	**-**	**-**	Thorn et al. (2020)
*Gy. dunensis*	Hum-46	Pakistan	MK088249	**-**	**-**	**-**	**-**	**-**	R. Khurshid et al. (2023)
*Gy. dunensis*	L90	Pakistan	MK088248	**-**	**-**	**-**	**-**	**-**	R. Khurshid et al. (2023)
*Gy. dunensis*	L04	Pakistan	MK088247	**-**	**-**	**-**	**-**	**-**	R. Khurshid et al. (2023)
*Gy. rubellus*	LAH36995	Pakistan	OL964420	OL964421	-	-	-	-	R. Khurshid et al. (2023)
*Gy. rubellus*	LAH36996	Pakistan	OL964403	OL964404	**-**	**-**	**-**	**-**	R. Khurshid et al. (2023)
*Gy. crociphyllus*	TNS-F-61956	Japan	KT368675	**-**	**-**	**-**	**-**	**-**	Kasuya et al. (2016)
*Gy. crociphyllus*	TNS-F-61966	Japan	KT368679	**-**	**-**	**-**	**-**	**-**	Kasuya et al. (2016)
*Gy. crociphyllus*	INM-2-87471	Japan	KU727211	**-**	**-**	**-**	**-**	**-**	Kasuya et al. (2016)
*Gy. crociphyllus*	INM-2-87449	Japan	KU727210	**-**	**-**	**-**	**-**	**-**	Kasuya et al. (2016)
*Gy. spectabilis*	TNS-F-61962	Japan	KT368688	**-**	**-**	**-**	**-**	**-**	Kasuya et al. (2016)
*Gy. purpureosquamulosus*	IBUG-89-16	Switzerland	AY280998	**-**	**-**	**-**	**-**	**-**	R. Khurshid et al. (2023)
*Gy. purpureosquamulosus*	K(M) 75214	Nigeria	AY280979	**-**	**-**	**-**	**-**	**-**	Liu and Bau (2019)
*Gy. bellulus*	TENN 069859	USA	KY744149	**-**	**-**	**-**	**-**	**-**	Liu and Bau (2019)
*Gy. bellulus*	SMNS-STU-F-0900398	Germany	MF039254	**-**	**-**	**-**	**-**	**-**	Eberhardt et al. (2018)
*Gy. austropicreus*	OTA 60208	New Zealand	OQ064819	**-**	**-**	**-**	**-**	**-**	Beaumont et al. (2002)
*Gy. austropicreus*	OTA 70412	New Zealand	OQ064892	**-**	**-**	**-**	**-**	**-**	Beaumont et al. (2002)
*Galerina marginata*	LE-BIN-2272	Russia	KY327302	**-**	**-**	**-**	**-**	**-**	Liu and Bau (2019)
*Ga. marginata*	IBUG-5246	Mexico	AY281020	**-**	**-**	**-**	**-**	**-**	R. Khurshid et al. (2023)

The bolded parts are the sequences generated in this study.

## Data Availability

All sequence data are available in NCBI GenBank following the accession numbers in the manuscript.
